# A Myriad of Symptoms After Spinal Anesthesia: A Case Report of Local Anesthetic Systemic Toxicity

**DOI:** 10.7759/cureus.29902

**Published:** 2022-10-04

**Authors:** Pedro Lavado, Eduardo Carvalho, Manuel Almeida, Isabel Taveira, Fernando Pádua

**Affiliations:** 1 Intensive Care Unit, Centro Hospitalar Universitário do Algarve, Portimão, PRT; 2 Intensive Care Unit, North Alentejo Local Health Unit, Portalegre, PRT

**Keywords:** last, spinal anesthesia, seizure, toxicity, local anesthetic, cardiac arrest

## Abstract

Local anesthetics are widely used by various medical professionals. Although their usefulness is unquestionable, as with any medication, there is a possibility of iatrogenic effects. When local anesthetic systemic toxicity occurs, it might be a life-threatening condition. Knowing its existence and how to act when it arises is crucial. The clinical presentation is wide-ranging, but globally it affects the neurological and cardiovascular system, with cardiac arrest being the extreme of its presentation. The treatment is mainly supportive with an attempt to reverse the effects of the anesthetic by administering a lipid emulsion. Here, we present a clinical case of difficult management with many complications.

## Introduction

The use of local anesthetics is a common practice in a variety of contexts by various medical specialties. As with any other drug, their use is not without side effects or toxicity. Local anesthetic systemic toxicity (LAST) may occur with all local anesthetics, despite their route of administration. Although rare, it may be a life-threatening condition, and specific management and awareness are fundamental. Here, we present a case in which the central nervous system (CNS) and cardiovascular system were affected.

## Case presentation

A 62-year-old woman was admitted for an elective knee replacement because of severe chronic osteoarthritis. Her medical history was unremarkable, with no current medication except painkillers. The preoperative assessment was normal, and the coronavirus disease 2019 test was negative.

A decision for spinal anesthesia was made, and the lumbar puncture was performed without complications. A total of 11.25 mg of bupivacaine and 3.75 µg of sufentanil were administered. A tremor was noted that retrospectively could have been the onset of neuroexcitability. Nonetheless, the procedure was continued after conversion to total intravenous (IV) anesthesia (propofol, fentanyl, and initial bolus of rocuronium) because spinal anesthesia was insufficient as the patient maintained local sensitivity. The endpoint of the bispectral index (BIS) values was 40-60. During the surgery, the patient was hypertensive and tachycardic, and esmolol was administered with effect.

After the suspension of IV anesthesia, the patient suffered a tonic-clonic seizure. At this time, it was suspected to be a case of LAST, in the absence of another etiology (no inhalational agents or succinylcholine) and possible clinical onset soon after the administration of local anesthetic. A lipid emulsion infusion was started (100 mL in three minutes and then 250 mL in 20 minutes). The seizure was first managed with propofol and midazolam, and the patient was admitted to the intensive care unit (ICU). Computed tomography (CT) of the head was normal.

In the first 10 hours in the ICU, the patient initially presented with hypertension (maximum 230/130 mmHg) which was managed with labetalol (max 2 mg/minute by continuous IV infusion). However, it later progressed to shock, requiring volume resuscitation (20 mL/kg in two hours), and norepinephrine perfusion was started as soon as volume resuscitation failed (max 2.5 µg/kg/minute). At this time, lipid emulsion was again administered to complete a total of 12 mL/kg. Despite these measures, refractory shock was seen, and epinephrine was started along with norepinephrine. An episode of ventricular tachycardia with pulse rapidly evolved to ventricular fibrillation and a return of spontaneous circulation was achieved after 10 minutes of advanced life support. Myocardial infarction was excluded.

In the first 24 hours, lactate was progressively higher with a maximum of 15 mmol/L despite a mean arterial pressure of >65 mmHg. An echocardiogram at this time showed a left ventricular dysfunction with an ejection fraction of 30-35%. Hemodynamic monitoring with PiCCO® (Pulse index Continuous Cardiac Output) was started and showed low cardiac and global end-diastolic indexes. After preload normalization, the cardiac index was persistently low, and dobutamine was initiated, admitting not only a distributive shock but also cardiogenic (Table 1).

**Table 1:** PiCCO®: hemodynamic monitoring. ↓: below normal range; ↑: above normal range; ↔: within normal range

Parameters	Initial with norepinephrine and epinephrine	After dobutamine
Global end-diastolic index (mL/m^2^)	518 ↓	721 ↔
Intrathoracic blood index (mL/m^2^)	648 ↓	798 ↓
Systemic vascular resistance index (Dynes second/cm^5^)	3273 ↑	1471 ↓
Cardiac index (L/minute/m^2^)	2.1 ↓	3.9 ↔
Global ejection fraction (%)	13 ↓	27 ↔
Extravascular lung water index (mL/kg)	13 ↑	12 ↑

Normalization of lactate was seen in 48 hours. Dobutamine was stopped on day six and norepinephrine the next day.

**Figure 1:**
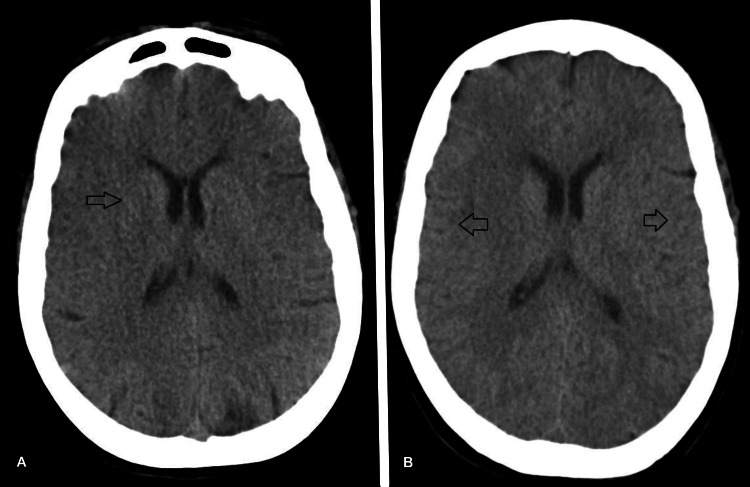
(A) Head CT on day four. (B) Head CT on day 12. Both CT scans show loss of the normal differentiation between cortical gray matter and subcortical white matter. CT: computed tomography

During the first 48-72 hours of ICU admission, the patient presented with episodic tonic-clonic seizures. Levetiracetam and phenytoin were started after neurology consultation, achieving complete seizure control. No electroencephalogram was performed because it was not available at the hospital. Head CT was repeated with incipient hypoxic-ischemic brain injury (Figure 1). Magnetic resonance imaging confirmed the presence of those lesions.

Despite difficult management with limited neurological monitoring, the patient had a favorable evolution and was discharged to the ward after 18 days.

After four months, the patient had only mild cognitive impairment (assessed by the Mini-Mental State Examination) as well as generalized muscular strength of four out of five, most likely due to ICU-acquired weakness.

## Discussion

The incidence of LAST is very low, with overall incidence ranging from 0.87 to 1.8 per 1,000 individuals [1,2]. Major events are seen in approximately 20% of the cases. Many minor events probably go unnoticed and unreported. Since the 80s, major events have decreased secondary to recognition and preventive measures [3]. Despite the low incidence, there is potential for severe toxicity. Knowledge about this entity is still lacking, as reported [4].

Local anesthetics block pain mainly by preventing sodium influx into the axon. The chemical structure is similar, but pharmacodynamic and pharmacokinetic properties change if they are racemic mixtures or pure enantiomers [5]. Bupivacaine, the drug used in our patient, is a racemic mixture and has a higher potential for cardiac and CNS toxicity compared with ropivacaine or levobupivacaine [6].

Toxicity presents when local anesthetics are absorbed or erroneously administered intravascularly and reach cardiac sodium channels [7] or thalamocortical neurons [8]. The classical presentation is characterized by the following progression soon after administration: CNS excitation, CNS inhibition, cardiovascular excitation, and, in extreme cases, cardiac arrest. Recent reviews have demonstrated not only a variable presentation but also a wide temporal range till the onset of symptoms [9,10]. Cardiac and CNS toxicity relates to anesthetic potency, dose, routes of administration, and injection techniques.

There are several risk factors for LAST, such as age extremes, cardiac disease, renal insufficiency, hepatic disease, pregnancy, carnitine deficiency, and block site [10].

As soon as LAST is recognized, the following steps should be taken for successful management: stop the injection if possible, provide the needed organ support, and administer IV lipid emulsion. After stabilization, the patients should be transferred to an ICU.

Not accounting for organ support, the management rests in the administration of IV lipid emulsion as soon as seizures or cardiovascular toxicity initiates. It seems to act in a multimodal way [11]: (1) removing the anesthetics from the heart and brain mainly to the liver [12]; and (2) possible cardiotonic effect [13] related to changes in fatty acid processing [14,15].

IV lipid emulsion is a 20% lipid emulsion. An initial bolus of 100 mL or 1.5 mL/kg should be given in three minutes followed by an infusion at 0.25 mL/kg/minute. The bolus can be repeated if cardiovascular stability is not achieved. The maximum total dose is 12 mL/kg [10,16].

The differential diagnosis for LAST should include thyroid storm, pheochromocytoma, malignant hyperthermia, anaphylaxis, and illicit drug toxicity. All of these were excluded in our case.

## Conclusions

Despite potentially life-threatening complications, the use of local anesthetics is essential. The identification of risk factors and prevention protocols may prevent its occurrence. Although rare, LAST exists, and knowledge of its clinical presentation and the initial treatment should be widespread. Clinical presentation is diverse and might prompt organ support. Although clinical presentation is variable, most commonly, neurological and cardiac dysfunction are the first to be seen.

Recognition and prompt treatment with the administration of IV lipid emulsion is recommended. Addressing hypoxia, acidosis, and seizures as well as providing cardiovascular support is needed in more severe cases. In this setting, ICU transfer is mandatory. Outcomes depend on a lot of factors, but a full recovery can be expected.
